# Acute intestinal obstruction caused by multifocal nodular inflammatory disease of the small intestine: a case report and literature review

**DOI:** 10.1186/s12893-026-03513-y

**Published:** 2026-01-21

**Authors:** Xirang Wang, Jian Kang, Yuxiang Li, Jing Fu, Xiaofeng Sun

**Affiliations:** 1 Department of General Surgery, Beijing Fengtai Youanmen Hospital, Beijing, 100069 China; 2 Department of General Surgery, Beijing Huimin Hospital, Beijing, 100054 China; 3 Department of Pathology, Beijing Fengtai Youanmen Hospital, Beijing, 100069 China

**Keywords:** Multifocal nodules, Small bowel obstruction, CMUSE, Case report

## Abstract

**Background:**

Multifocal nodular inflammatory disease of the small intestine is a rare cause of acute intestinal obstruction. We report such a case to discuss its diagnostic approach, emphasizing the role of surgical intervention and pathological examination, thereby aiming to improve clinical recognition of this condition.

**Clinical presentation:**

We analyzed the clinical data of a 33-year-old male patient who was admitted with intermittent abdominal pain and distension for one week, which aggravated over one day. Emergency computed tomography (CT) revealed obstruction of the proximal jejunum with inflammatory changes, raising suspicion of internal hernia. Diagnostic laparoscopy confirmed the absence of internal hernia and volvulus, and no bowel resection was performed. Multiple nodules were observed throughout the small intestine, accompanied by thickened and edematous intestinal wall at the site of obstruction in the proximal small bowel. One intestinal nodule was biopsied during surgery for pathological examination. Histopathological findings indicated chronic mucosal inflammation, lymphoid follicular hyperplasia, superficial ulceration with acute inflammatory cell infiltration, extensive edema, and focal hemorrhagic changes. Postoperative management included anti-inflammatory therapy and nutritional support. The patient recovered well and was discharged. During a 9-month follow-up, he remained asymptomatic with normal dietary intake and bowel function, without recurrence of the disease.

**Conclusion:**

This case of multifocal nodular inflammatory disease of the small intestine was most consistent with Cryptogenic Multifocal Ulcerating Stenosing Enteropathy (CMUSE), a rare inflammatory disorder characterized by multiple superficial ulcers and stenotic lesions in the small intestine. Although rare, CMUSE can lead to intestinal obstruction. Diagnostic laparoscopy and pathological biopsy are crucial for its diagnosis.

## Background

Intestinal obstruction comprises 2%–8% of acute abdomen cases presenting to emergency departments. Approximately 80% of these cases involve obstruction of the small bowel. The most common causes of intestinal obstruction are postoperative adhesions, hernias, and tumors [1]. Small bowel obstruction (SBO) with multiple nodular lesions require differentiation from various conditions, including small intestinal neoplasms (such as small intestinal neuroendocrine tumors [SI-NETs], lymphoma, and gastrointestinal stromal tumors [GISTs]), inflammatory bowel diseases (e.g., Crohn’s disease and intestinal tuberculosis), drug-induced enteropathy, and rare disorders like CMUSE [2–4].

CMUSE is an exceptionally rare disease specific to the small intestine, with most reported cases being isolated. It is characterized by multiple superficial ulcers and stenotic lesions, which can lead to recurrent abdominal pain, anemia, or acute intestinal obstruction [2]. The prognosis is relatively favorable, and early surgical intervention for strangulating obstruction can prevent bowel necrosis and perforation. Delayed diagnosis, however, may result in progression to bowel necrosis and systemic infection [5]. Therefore, while CMUSE is rare, it represents an important diagnostic consideration in cases of unexplained, acute small bowel obstruction—particularly in younger patients. The present case is reported to highlight that a definitive diagnosis often hinges on laparoscopic exploration and histological confirmation, which can prevent unnecessary extensive resection. For surgical practice, this underscores a critical management principle: in the setting of acute obstruction with multifocal nodules, intraoperative biopsy should be prioritized to distinguish this inflammatory condition from neoplastic processes, thereby directly guiding appropriate intervention.

## Case presentation

A 33-year-old male presented to the emergency department with a one-week history of intermittent abdominal pain and distension that had worsened over the previous 24 h. The abdominal pain, described as colicky and predominantly localized in the epigastric region, was accompanied by distension, nausea. He initially sought care at a local hospital where a diagnosis of intestinal obstruction was made, and supportive management was initiated. One day prior to admission, his symptoms aggravated with persistent pain and vomiting of gastric content. He was referred to our hospital for further management. Upon emergency evaluation, he was admitted to our department with suspected intestinal obstruction and possible internal hernia. Since the onset of symptoms, the patient had been unable to tolerate oral intake and had not passed any stool. His past medical history was unremarkable, with no reported family history of hereditary diseases.

### Physical examination on admission

The abdomen was distended with muscular rigidity. No hepatosplenomegaly was detected. Diffuse tenderness and rebound tenderness were observed, particularly pronounced in the upper abdomen. No shifting dullness was noted. Bowel sounds were diminished.

### Ancillary investigations

**Fig. 1:**
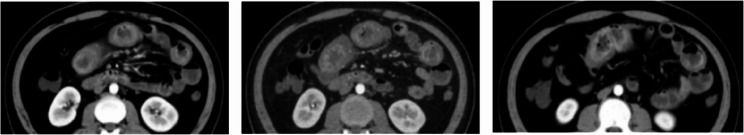
Preoperative contrast-enhanced CT findings. Imaging revealed a proximal jejunal obstruction with inflammatory wall changes, minimal adjacent exudate, and localized peritonitis. The presence of mesenteric congestion and distortion in the upper abdomen suggested the possibility of an internal hernia

An enhanced abdominal CT scan (Fig. 1) demonstrated obstruction of the proximal jejunum with inflammatory changes, minimal surrounding exudation, and congestion and distortion of the mesentery in the upper abdomen, raising suspicion of internal hernia.

### Laboratory tests showed

White blood cell count 16.95 (reference values3.50-9.50.50) ×10^9^/L, absolute neutrophil count 14.10 (reference values1.80-6.30.30) ×10^9^/L, neutrophils 83.2% (reference values40.0%−75.0%), hemoglobin 162 (reference values130-175) g/L, platelet count 337 (reference values125-350) ×10^9^/L, C-reactive protein 133.32 (reference values0.00–6.00.00) mg/L, interleukin-6 400.12 (reference values ≤ 7.00) pg/mL, procalcitonin 5.32 (reference values < 0.50) ng/mL, blood lactate 1.47 (reference values 0.00–1.30.00.30) mmol/L, albumin 35.3 (reference values 35.0–50.0) g/L, plasma D-dimer 6.85 (reference values 0.00–0.55.00.55) mg/L, and fibrinogen 5.04 (reference values 2.00–4.00) g/L.

### Admission diagnosis

Acute peritonitis and strangulated intestinal obstruction.

### Hospital course

**Fig. 2:**
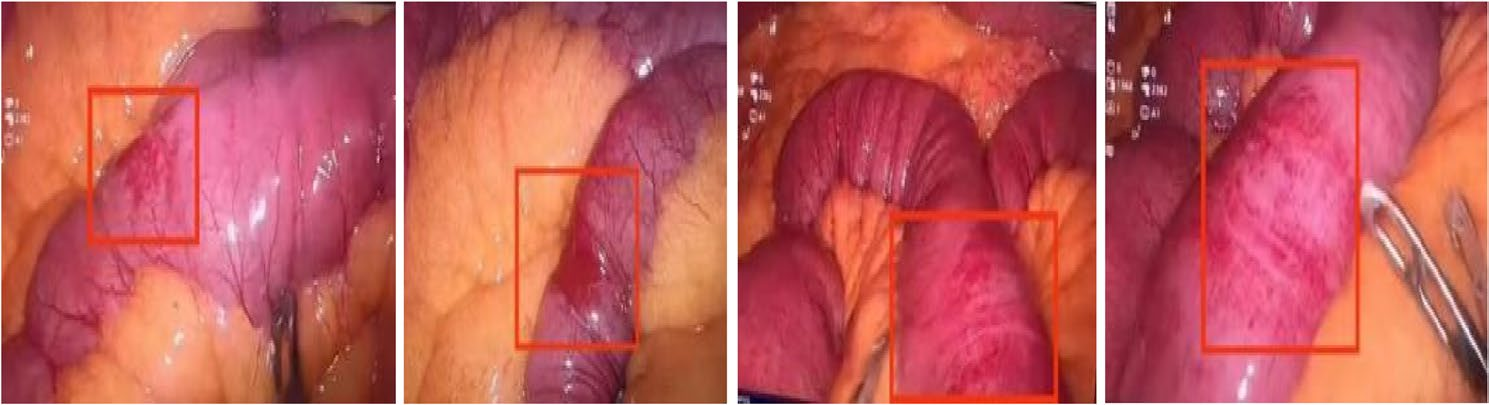
Intraoperative view of nodules in the distal non-obstructed segment of the small intestine

Based on the clinical history, signs of peritonitis, contrast-enhanced abdominal CT findings, and laboratory results, internal hernia with possible strangulated small bowel obstruction was suspected. Nasogastric decompression was initiated. After discussion with the patient and his family, emergency diagnostic laparoscopy was performed. Intraoperatively, a small amount of blood-tinged ascitic fluid was found in the abdominopelvic cavity. The proximal small bowel was significantly dilated with wall thickening, edema, and dark reddish discoloration. No hernia ring, entrapment marks on the small bowel or mesentery, or volvulus were identified. Multiple nodules were scattered throughout the entire small intestine, most prominently in the proximal segment. The serosal surface over these nodules showed hypervascular congestion centered on the nodules, with localized wall thickening. The bowel wall between nodules in the distal segment appeared normal (Fig. 2). Intraoperative assessment favored an inflammatory process, though malignancy could not be entirely excluded. The proximal SBO and ischemic changes were attributed to these multiple nodules, causing wall thickening, luminal stenosis, and subsequent obstruction with ischemia. Given that weak peristalsis was still present and the ischemic bowel had not yet progressed to necrosis, and after thorough communication with the family, bowel resection was deferred. A biopsy of one nodule from the dilated segment was performed with informed consent.

### Histopathological findings (Fig. 3)

The biopsy showed chronic mucosal inflammation, localized lymphoid follicular hyperplasia, and areas of ulceration confined to the mucosa. There was infiltration by numerous acute inflammatory cells, extensive edema, vascular dilation and congestion, and focal hemorrhagic changes.

**Fig. 3:**
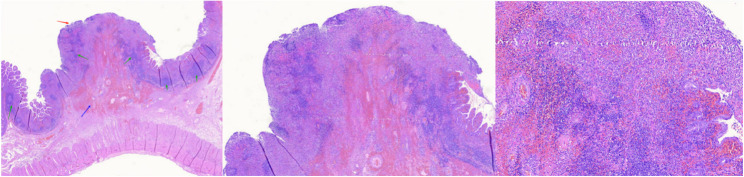
Histopathological findings of the small intestinal nodule biopsy. Histopathological examination revealed: focal hemorrhage, edema, and thickening of the intestinal submucosa (blue arrow); loss of surface epithelium with formation of superficial ulcerations (red arrow); and lymphoid hyperplasia (green arrow). The lamina propria was infiltrated by inflammatory cells, including lymphocytes, mononuclear cells, and neutrophils. The inflammation extended into the submucosal layer.

### Postoperative management and outcome

The patient received postoperative antibiotic therapy and nutritional support, including nasogastric decompression. A gradual oral diet was reintroduced starting one week after surgery. During a 9-month follow-up, the patient reported normal dietary intake, regular bowel movements, and no recurrence of symptoms.

#### Discussion and conclusions

The pathogenesis of CMUSE remains unclear, though associations with immune dysregulation or genetic mutations (e.g., SLCO2A1) have been suggested [2, 4]. Pathologically, CMUSE is characterized by recurrent mucosal ulceration and healing, leading to stenosis and obstruction, typically in the proximal or mid‑small intestine. Histology shows chronic mucosal inflammation, lymphoid follicular hyperplasia, and superficial ulcers. A limitation in the present case is that immunohistochemical analysis was not performed.

CMUSE may present with recurrent abdominal pain, anemia, or acute intestinal obstruction [2]. Its clinical picture requires differentiation from other multifocal small‑bowel disorders. Neoplastic lesions include small intestinal neuroendocrine tumors (SI‑NETs), which often occur in the terminal ileum, are frequently multifocal [3], and may cause abdominal pain, bleeding, or hormonal syndromes [6, 7]; lymphoma, which commonly involves the distal ileum and can present with pain, bleeding, anemia, or obstruction [8, 9]; and gastrointestinal stromal tumors (GISTs), most frequent in the stomach but also occurring in the small intestine, associated with abdominal pain or bleeding [10, 11]. Inflammatory and other conditions comprise Crohn’s disease, manifesting as chronic abdominal pain, diarrhea, obstruction, or perianal complications [12, 13]; intestinal tuberculosis, which typically presents with chronic pain, low‑grade fever, night sweats, weight loss, and may lead to obstruction or bleeding [14, 15]; and drug‑induced enteropathy (e.g., NSAID‑related), whose symptoms often reverse after drug cessation [2, 16].

Preoperative CT can demonstrate obstruction and inflammatory changes but often fails to identify multifocal lesions or distinguish benign from malignant etiologies. In acute strangulated obstruction with high‑risk features (e.g., peritonitis, elevated lactate), emergency surgery is imperative [17]. Direct intraoperative exploration and biopsy provide definitive diagnostic evidence and reduce the risk of bowel necrosis [18]; early surgical intervention is superior to non‑operative management in such high‑risk patients [19]. In this case, pathological examination ruled out neoplastic conditions: SI‑NETs typically show solid neuroendocrine cell proliferation expressing SSTR‑2 and serotonin‑secreting EC cells [20, 21]; lymphoma displays glandular atrophy, diffuse infiltration of small lymphocytes, increased intraepithelial lymphocytes, villous atrophy, and paucity of lamina propria plasma cells [22]; GISTs exhibit KIT/PDGFRA mutations, spindle‑cell morphology, and CD117/DOG‑1 positivity [23, 24]. Crohn’s disease is characterized by transmural inflammation, non‑caseating granulomas, and fissuring ulcers [25]; intestinal tuberculosis by caseating granulomas, sometimes acid‑fast stain positive [15]; and drug‑induced enteropathy by nonspecific mucosal inflammation, erosions, or ulcers [16]. The surgical and pathological findings were most consistent with CMUSE.

For acute strangulating obstruction, timely surgical intervention is crucial to establish a diagnosis and prevent bowel necrosis. Postoperatively, our patient received no specific medication and remained asymptomatic during 9‑month follow‑up, although disease recurrence remains a concern [26]. For refractory cases, immunomodulatory therapies have been reported, including corticosteroids, mesalazine, and—in isolated severe cases—ustekinumab [4, 27]. Genetic testing (e.g., for SLCO2A1 mutations) is recommended in patients with refractory or recurrent ulcers, a family history, or consanguinity, to guide individualized management [28].

In conclusion, this case highlights that CMUSE, though rare, can present acutely as small bowel obstruction necessitating urgent surgery. Definitive diagnosis relies on laparoscopic exploration and histopathological biopsy of nodules, crucial for differentiating it from neoplasms. This underscores the importance of including CMUSE in the differential diagnosis of unexplained obstruction in young adults. A favorable outcome may be achieved with timely intervention, and refractory cases may warrant corticosteroids and immunomodulatory therapy.

## Data Availability

The datasets used and/or analyzed during the current study are available from the corresponding author on reasonable request.

## Abbreviations

CT: Computed tomography
CMUSE: Cryptogenic Multifocal Ulcerating Stenosing Enteropathy
SBO: Small bowel obstruction
SI-NETs: Small intestinal neuroendocrine tumors
GISTs: Gastrointestinal stromal tumors
EC: Enterochromaffin
